# Diagnostic value of anti-topoisomerase I antibodies in a large monocentric cohort

**DOI:** 10.1186/ar2622

**Published:** 2009-02-21

**Authors:** Katharina Hanke, Cornelia Dähnrich, Claudia S Brückner, Dörte Huscher, Mike Becker, Anthonina Jansen, Wolfgang Meyer, Karl Egerer, Falk Hiepe, Gerd R Burmester, Wolfgang Schlumberger, Gabriela Riemekasten

**Affiliations:** 1 Department of Rheumatology and Clinical Immunology, Charité Universitätsmedizin Berlin, Charitéplatz 1, Berlin 10117, Germany; 2 EUROIMMUN Medizinische Labordiagnostika AG, Seekamp 31, Lübeck 23560, Germany; 3 German Rheumatism Research Centre, Charitéplatz 1, Berlin 10117, Germany

## Abstract

**Introduction:**

In the present study, the detection of anti-topoisomerase I (anti-topo I) autoantibodies was evaluated for diagnosis and risk assessment of systemic sclerosis (SSc) patients in a well characterized large monocentric cohort.

**Methods:**

Sera from patients with SSc (diffuse n = 96, limited n = 113), from patients with overlap syndromes (n = 51), from patients with other diseases associated with SSc (n = 20), as well as from disease controls (n = 487) were analysed for the presence of anti-topo I antibodies by line immunoblot assay and ELISA. Assessment of organ manifestations was performed as proposed by the European Scleroderma Trial and Research network.

**Results:**

The applied test systems for the detection of anti-topo I antibodies revealed a diagnostic sensitivity for SSc of approximately 24% and a diagnostic specificity of at least 99.6%. The sensitivity to identify patients with diffuse SSc amounted to 60%. Patients with anti-topo I antibodies showed a higher burden of skin and lung fibrosis, contractures, electrocardiogram changes, as well as digital ulcers and had more active disease than antibody-negative patients. Signal strengths correlated only weakly with disease activity, with modified Rodnan skin score, with predicted forced vital capacity, and with predicted diffusion capacity levels (*P *= 0.01, ρ = 0.234, ρ = 0.413, ρ = -0.215, ρ = -0.219). High signal intensities were associated with an increased mortality in diffuse SSc patients (*P *= 0.003).

**Conclusions:**

Diagnosis and risk assessment of SSc patients can be supported by the detection of anti-topo I antibodies. Signal intensities as obtained by line immunoblot assay or ELISA can be used as a surrogate marker for fibrosis, active disease and worse prognosis.

## Introduction

Systemic sclerosis (SSc) is a rare and heterogeneous disease with different disease subsets. Its outcome may vary from mild to very severe, life-threatening disease rapidly leading to death. The detection of autoantibodies, especially directed towards topoisomerase I (anti-topo I), can help to identify patients at risk for progressive and severe disease and to classify them according to their disposition for certain clinical manifestations [1-6]. Depending on the studies, however, antibody frequencies varied between 14% [2] and 70% [4].

Several commercially available test systems can be used for the detection of anti-topo I antibodies. Among these systems, efficient monospecific methods – such as ELISAs and line immunoblot assays (LIAs) for single-parameter or profile analysis – have been established in clinical laboratory practice during past years, allowing examination of a large number of sera in a rapid approach at high sensitivity and at high specificity [7-10].

The precise clinical characterization of the patients and their assessment are crucial for the evaluation of a new assay or of a potential biomarker such as anti-topo I antibodies as well as for the comparison with other studies. As shown before, differences in the frequencies of anti-topo I antibodies in different studies might be caused by ethnic background [1,11] or by different classification systems of SSc [1,2,12-14]. Most studies have used the American College of Rheumatology classification criteria [1,2,13]; however, there are some limitations especially for early and probably very late cases, as these criteria were originally not intended to be used as a diagnostic tool. Other studies have divided their patients into diffuse, limited, and intermediate SSc patients or have included overlap syndromes [13,14]. In recent years, the LeRoy criteria for classification have been widely used. In this classification system, however, the presence of anti-topo I antibodies is often associated with diffuse SSc [15].

Most studies have shown that anti-topo I-positive patients suffer more frequently from severe lung and skin fibrosis [4,6,13,16]. Several studies have identified further associations not confirmed by others while using different assessment strategies to define organ involvement or disease activity [17,18]. Additionally, the interval between the detection of antibodies and the clinical assessment often remains unspecified [1]. National and multinational networks such as the European Systemic Sclerosis Trial and Research (EUSTAR) network or the German Network of Systemic Sclerosis (DNSS) addressed the standardization in the assessment and classification of patients. Despite these efforts, both intraobserver variability and interobserver variability are still significant, especially when multicentric studies are performed [19].

In the present cross-sectional monocentric study, we analysed a large cohort of genuine SSc patients, patients with diseases related to SSc as well as numerous disease controls for the presence and diagnostic impact of anti-topo I antibodies detected by means of commercially available, monospecific LIA and ELISA. In order to minimize limitations of former studies, clinical data were assessed simultaneously to antibody detection by a standardized procedure with only a limited number of investigators.

## Materials and methods

### Classification of patients

Sera from 280 consecutive patients with SSc were tested for the presence of anti-topo I antibodies. As disease controls we included serum samples from patients with myositis (n = 26), from patients with systemic lupus erythematosus (n = 208), from patients with Sjögren's syndrome (n = 88) and from patients with rheumatoid arthritis (n = 165). All patients were diagnosed at the Charité University of Medicine (Berlin, Germany).

Classification of SSc patients was performed before starting the study based on the maximal skin involvement during the disease course, on the basis of the LeRoy criteria and according to the EUSTAR and DNSS criteria [20]. Briefly, SSc patients were classified as having either limited SSc or diffuse SSc depending on the distribution of skin sclerosis below or above the elbows, knees or clavicles [15]. Once classified as having diffuse SSc, the patient remains classified as having diffuse SSc. Characteristics of SSc patients are presented in Table 1.

**Table 1:** Clinical and demographic characteristics of the systemic sclerosis (SSc) cohort (Charité University)

	Diffuse SSc	Limited SSc	SSc sine scleroderma	Overlap	Mixed connective tissue disease	Undifferentiated connective tissue disease	All patients
Anti-topoisomerase I (LIA)	58 (60.4)	7 (6.2)	1 (25.0)	1 (2.6)	0	0	67 (23.9)
Anti-topoisomerase I (ELISA)	57 (59.4)	6 (5.3)	1 (25.0)	1 (2.6)	0	0	65 (23.2)
Number	96	113	4	38	13	16	280
Age (years)	53 ± 14.5	60 ± 11.3	54 ± 12.7	52 ± 13.3	52 ± 13.5	56 ± 10.7	56 ± 13.2
Duration of Raynaud's phenomenon (years)	9 ± 10.6	14 ± 13.6	18 ± 18.47	8 ± 7.4	16 ± 10.4	11 ± 9.3	12 ± 11. 6
Duration of non-Raynaud's phenomenon symptoms	7.8 ± 1.6	9.5 ± 1.39	6 ± 10.8	8.8 ± 2.2	9.1 ± 5.5	9.3 ± 6.3	8.7 ± 7.6
Duration since disease diagnosis (years)	6 ± 7.38	8 ± 7.4	3 ± 2.6	6 ± 5.17	9.1 ± 5.5	7 ± 7.28	7 ± 7.38
Female/male	79/17	105/8	2/2	28/10	13/0	16/0	243/37
mRSS	13.2 ± 9.6	5.2 ± 4.2	1.5 ± 1.1	6.7 ± 7.5	1.6 ± 1.8	0.8 ± 1.1	7.6 ± 8.0
Digital ulcers	52 (54.2)	38 (33.6)	2 (50)	14 (36.8)	4 (30.8)	2 (12.5)	112 (40.0)
Lung fibrosis	57 (59.4)	16 (14.2)	3 (75)	16 (42.1)	4 (30.8)	2 (12.5)	98 (35.0)
DLCO by a single breath (%)	64.9 ± 21.9	76,8 ± 17.5	56.6 ± 12.8	64.2 ± 22.4	75.3 ± 17.8	75.4 ± 17.1	70.5 ± 20.5
Mean FVC (%)	81.7 ± 18.93	96.7 ± 15.2	87.5 ± 30.7	84.0 ± 19.2	92.9 ± 23.5	92 ± 18.0	89,2 ± 19.
Contractures	78 (81.3)	62 (54.9)	2 (50)	23 (60.5)	4 (30.8)	3 (18.8)	172 (61.4)
Pulmonary arterial hypertension	22 (22.9)	22 (19.5)	2 (50)	9 (23.7)	4 (30.8)	1 (6.3)	60 (21.4)
Renal involvement	17 (17.7)	22 (19.5)	1 (25)	10 (26.3)	4 (30.8)	2 (12.5)	56 (20)
Renal crisis	10 (10.4)	3 (2.7), n = 112	1 (25)	1 (2.6)	0	1 (6.3)	16 (5.7), n = 279
Cardiac involvement	47 (49.0)	35 (31.0)	3 (75)	19 (50)	6 (46.2)	4 (25)	117 (40.7)
Skin involvement	94 (98.9)	105 (92.9)	3 (75)	32 (84.2)	7 (53.8)	7 (43.8)	248 (88.9)
Raynaud's phenomenon	95 (99)	110 (97.3)	4 (100)	38 (100)	12 (92.3)	13 (81.3)	272 (97.1)

Data presented as mean ± standard deviation or *n *(%). DLCO, predicted diffusion capacity; FVC, predicted forced vital capacity; LIA, line immunoblot assay; mRSS, modified Rodnan Skin Score.

Overlap syndrome was defined as a disease occurring with clinical aspects of SSc (according to the American College of Rheumatology criteria) or with the main symptoms of SSc simultaneously with those of other connective tissue diseases/other autoimmune diseases, such as dermatomyositis, Sjögren's syndrome or systemic lupus erythematosus. These patients are mostly positive for anti-U1-RNP antibodies or anti-PM-Scl antibodies [21,22]. SSc sine scleroderma was defined by the lack of skin alterations in the presence of other SSc symptoms [23]. Undifferentiated connective tissue disease with scleroderma features was defined as positive Raynaud's phenomenon and at least one further feature of SSc and/or detectable scleroderma-associated autoantibodies [24].

### Assessment of systemic sclerosis patients

With the exception of the subclassification of SSc patients, clinical data were collected simultaneously at the time sera were obtained for the detection of anti-topo I antibodies. Most patients were assessed by one investigator (GR), and the second investigator involved (CB) was instructed by the first investigator. Both investigators participated in several training programs of EUSTAR and DNSS for the assessment of SSc patients.

The modified Rodnan Skin Score (mRSS) was used for the evaluation of fibrotic skin changes [25,26]. Cardiac involvement was defined by the presence of two of the following symptoms: diastolic dysfunction, conduction abnormalities, cardiomyopathy, or reduced ejection fraction unrelated to other diseases, valvular changes such as tricuspidal insufficiency not explained for by other causes than SSc, or pericarditis. Conduction blocks and signs of atrial and ventricular hypertrophy related to SSc were summarized as electrocardiogram changes. Disease activity was obtained using the EUSTAR activity index [17]. Pulmonary arterial hypertension was defined by a mean pulmonary arterial pressure of 25 mmHg at rest and 30 mmHg at exercise when assessed by right heart catheterization or by the presence of pulmonary artery systolic pressure ≥ 40 mmHg as detected by echocardiography and signs of right heart failure. Pulmonary fibrosis was diagnosed by chest radiogram and/or by high-resolution computed tomography scans. Lung function was assessed by the predicted forced vital capacity (FVC) and the predicted diffusion capacity (DLCO) by a single breath method. Renal involvement was diagnosed by present or past renal crisis or impaired kidney function unexplained by other causes. Digital ulcers were defined as a loss of both epidermis and dermis in an area of at least 2 mm diameter at the distal phalanx of fingers.

Patients were included in the study between January 2004 and May 2007. For a prospective survival analysis, patients were observed for a mean period of 24.6 months (18 to 52 months). To assess cumulative survival data from the time point of diagnosis, primary care doctors or patients were contacted in order to monitor survival and treatment in patients without regular appointments.

The study was approved by the local ethical committee (EA1/013/705). Written informed consent was obtained from each patient.

### Antibody detection

Sera from consecutive patients with SSc-related diseases as well as sera serving as disease controls were obtained simultaneously with the clinical assessment of the patients and were stored at -20°C. Anti-topo I antibodies were detected by commercially available, CE-certified monospecific LIA (Anti-Scl-70 EUROLine) and ELISA (Anti-Scl-70 ELISA) provided by EUROIMMUN AG (Lübeck, Germany). Blot strips and ELISA wells are coated with commercial Scl-70 antigen purified by affinity chromatography from bovine thymus (>90% purity). This antigen (molecular weight 75 to 82 kDa) represents a proteolytic degradation product of the predominant *in vivo *form of the enzyme DNA topoisomerase I (molecular weight 100 kDa); both the native and lower molecular weight forms have been shown to effectively bind autoantibodies against topoisomerase I [27]. Antigen reactivity of LIA and ELISA was verified using the human reference serum CDC-ANA #9 (Center for Disease Control, Atlanta, GA, USA) and both test systems were validated thoroughly using well characterized positive and negative controls.

All analyses were performed according to the manufacturer's instructions and were carried out blindly by personnel unaware of the diagnosis and the clinical characteristics of the patients.

For the LIA, human sera were diluted 1:101 prior to use and antibody detection was performed using alkaline phosphatase-labelled goat anti-human IgG. Control sera were included in each assay. Incubated blot strips were digitalized using a flatbed scanner. The intensity of the bands was automatically evaluated by a computer program. Signal strengths above 6 units were considered positive, as recommended by the manufacturer.

With respect to the ELISA, all serum samples were analysed at a dilution of 1:201 in parallel with control sera. Peroxidase-labelled rabbit anti-human IgG served as the secondary antibody conjugate. For evaluation, data above the cut-off value of 20 units/ml were considered positive, complying with the manufacturer's recommendation.

### Statistical analysis

The dataset was analysed using the SPSS V 15.0 statistical package (NASDAQ, Bloomingdale, IL 60108, USA) and the Microsoft calculation software Excel V 12 (2007; Microsoft corporation, USA). To identify associations between SSc symptoms and the occurrence of anti-topo I antibodies, chi-square tests, Mann–Whitney U tests, and Wilcoxon signed-rank tests were performed when appropriate. Spearman's rank correlation coefficient (ρ) was applied to analyse possible correlations. For all tests, *P *< 0.05 was considered statistically significant.

## Results

### Diagnostic sensitivity and specificity of anti-topoisomerase I antibody detection and identification of SSc patients with diffuse disease

**Figure 1:**
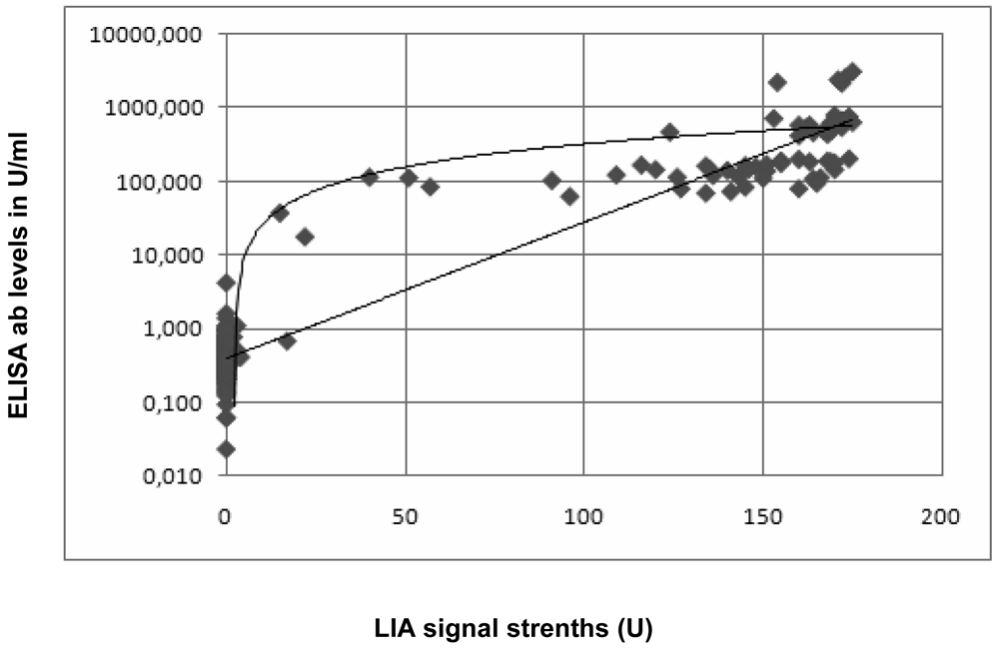
Line immunoblot assay signal strengths and logarithmized ELISA values to detect anti-topoisomerase I antibodies. Correlation between line immunoblot assay (LIA) signal strengths and logarithmized ELISA values for the detection of anti-topoisomerase I antibodies in our cohort of 280 patients. The logarithmic curve as indicated by the Spearman correlation coefficient reflects the correlation better than a linear correlation (as shown by the line). ab, antibody; U, units.

Using the monocentric SSc cohort (n = 280) and four disease control cohorts (n = 487), the detection of anti-topo I antibodies revealed a diagnostic sensitivity for SSc of 23.9% (23.2%) and a diagnostic specificity of 99.6% (99.8%), as determined by LIA (ELISA) (Table 2). In general, a significant correlation was observed between LIA signal strengths and antibody levels as detected by ELISA (*P *< 0.0005, ρ = 0.752; Figure 1).

**Table 2:** Sensitivity and specificity of anti-topoisomerase I antibodies

Panel	*n*	Line immunoblot assay	ELISA
Diffuse systemic sclerosis^a^	96	58 (60.4%)	57 (59.4)
Limited systemic sclerosis^a^	113	7 (6.2%)	6 (5.3%)
Systemic sclerosis sine scleroderma^a^	4	1 (25%)	1 (25%)
Overlap^a^	38	1 (2.6%)	1 (2.6%)
Mixed connective tissue disease^a^	13	0	0
Undifferentiated connective tissue disease^a^	16	0	0
Sensitivity^a^	280	67 (23.9%)	65 (23.2%)
Myositis^b^	26	0 (100%)	0 (100%)
Systemic lupus erythematosus^b^	208	0 (100%)	0 (100%)
Sjögren's syndrome^b^	88	0 (100%)	0 (100%)
Rheumatoid arthritis^b^	165	2 (98.8%)	1 (99.4%)
Specificity^b^	487	2 (99.6%)	1 (99.8%)

^a^Data presented as number of patients positive in the respective test (percentage within the respective patient cohort). ^b^Data presented as number of patients positive in the respective test (percentage of negative results within the respective control cohort).

Since the SSc cohort was rather heterogeneous, the frequency of anti-topo I antibodies was determined for the distinct patient groups and disease manifestations, resulting in sensitivities and specificities for the serological identification of these clinical characteristics with reference to the SSc cohort (Table 2 and Table 3). LIA analyses revealed the presence of anti-topo I antibodies in 58 (60.4%) out of 96 patients with diffuse SSc. In contrast, only seven (6.2%) out of 113 patients with limited SSc, just one among 38 patients with an overlap syndrome other than mixed connective tissue disease and only one out of four patients with SSc sine scleroderma were positive for anti-topo I antibodies. Identical results were obtained by means of ELISA, with the exception of only 57 (59.4%) anti-topo I-positive patients with diffuse SSc and six positive results (5.3%) in the group of limited SSc. Based on these data, detection of anti-topo I antibodies by means of LIA (ELISA) revealed a sensitivity for diffuse SSc of 60.4% (59.4%) with a specificity of 95.1% (95.6%), referring to the cohort of 280 patients with SSc.

**Table 3:** Anti-topoisomerase I antibody-positive versus antibody-negative patients in systemic sclerosis cohort assessed by line immunoblot assay

Disease manifestation	Patients with disease manifestation	Anti-topoisomerase I		*P *value	Sensitivity (%)	Specificity (%)
					
		Positive (n = 67)	Negative (n = 213)			
Digital ulcers	112 (40.0%)	38 (56.7%)	74 (34.7%)	0.002	33.9	82.7
Lung fibrosis	98 (35.0%)	46 (68.6%)	52 (24.4%)	<0.0005	46.9	88.5
DLCO by a single breath		62.9 ± 20.8	72.9 ± 19.9	<0.0005	NA	NA
Mean FVC		81.1 ± 18.0	91.8 ± 18.7	<0.0005	NA	NA
Pulmonary arterial hypertension	60 (21.4%)	17 (25.4%)	43 (20.2%)	0.39	28.3	77.3
Contractures	162 (57.9%)	59 (88.1%)	113 (53.1%)	<0.0005	34.3	92.6
Renal involvement	56 (20.0%)	13 (19.4%)	43 (20.2%)	1	23.2	75.9
Renal crisis	16 (5.7%)	6 (9%)	10 (4.7%)	0.23	37.5	76.8
Cardiac involvement	114 (40.7%)	33 (49.2%)	81 (38.0%)	0.12	28.9	79.5
Conduction blocks	69 (24.6%)	25 (37.3%)	44 (20.7%)	0.009	36.2	80.1
Electrocardiogram changes	74 (26.4%)	27 (40.3%)	47 (22.1%)	0.007	36.5	80.4
mRSS		14.2 ± 10.2	5.57 ± 5.8	<0.0005	NA	NA
Raynaud's phenomenon	272 (97.1%)	67 (100%)	205 (96.2%)	0.2	24.6	100
Diffuse systemic sclerosis	96 (34.3%)	58 (86.6%)	38 (17.8%)	<0.0005	60.4	95.1
Mean disease activity		2.49	1.63	<0.0005	NA	NA

Data presented as *n *(%) or mean ± standard deviation. DLCO, predicted diffusion capacity; FVC, predicted forced vital capacity; mRSS, modified Rodnan skin score; NA, not applicable.

Moreover, LIA signal strengths were stronger in diffuse SSc patients compared with limited SSc patients. The mean signal intensity was 148.4 units (standard deviation = 4.4) in diffuse SSc patients and 103.6 units (standard deviation = 24.5) in limited SSc patients (*P *< 0.0005), supporting the quantitative data obtained by ELISA (data not shown). With the ELISA, patients with diffuse SSc had higher mean values (467.6 units/ml, standard deviation = 68.1) when compared with patients with limited SSc (233.2 units/ml, standard deviation = 97.3, *P *< 0.0005).

Owing to the substantial degree of diagnostic conformity between LIA and ELISA (reflected by the above presented data and the data not shown), only results obtained by LIA are presented in the following sections.

### Anti-topoisomerase I antibodies were associated with peripheral vascular complications but not with pulmonary arterial hypertension or renal crisis

Patients with anti-topo I antibodies revealed a higher frequency of present or past digital ulcers (Table 3): 57% of the anti-topo I-positive patients suffered from present or past digital ulcers. In patients without anti-topo I antibodies, the prevalence of digital ulcers was significantly lower (32.2%).

In contrast, there was no association between the presence of anti-topo I antibodies and pulmonary arterial hypertension or renal involvement including renal crisis. There was also no association between the presence of anti-topo I antibodies and neuropathies, heart (despite conduction disturbances) and gastrointestinal involvement, renal involvement, sicca syndrome, joint involvement, or any other clinical symptom assessed by the EUSTAR and DNSS core dataset (data not shown) [16,28].

### Anti-topoisomerase I antibodies characterize patients with fibrotic SSc features and active disease

**Figure 2:**
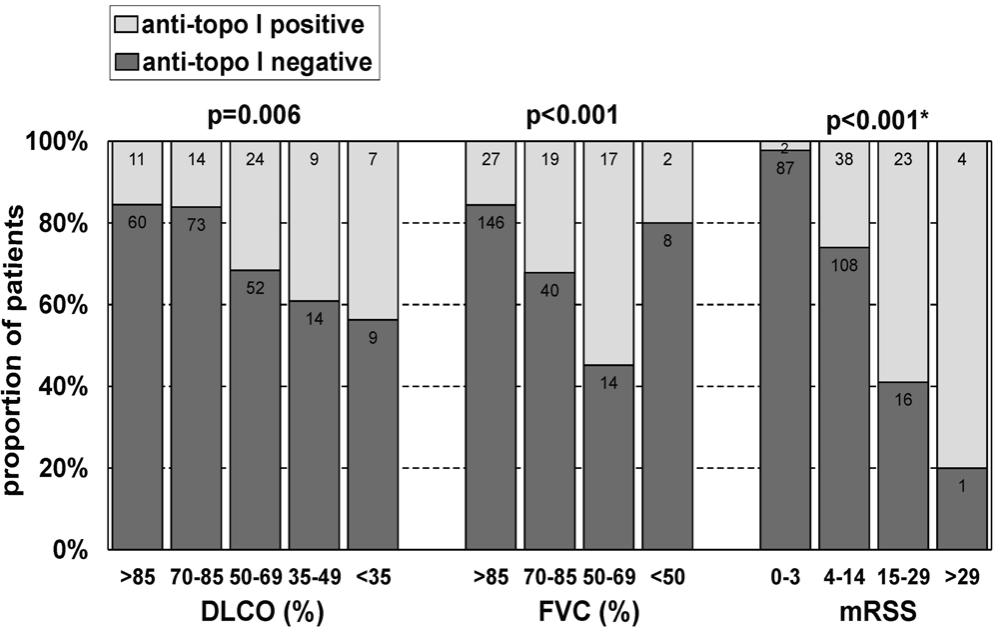
Detection of anti-topoisomerase I antibodies by line immunoblot assay. Detection of anti-topoisomerase I (anti-topo I) antibodies by line immunoblot assay with respect to different levels of predicted diffusion capacity (DLCO) by a single breath (%), predicted forced vital capacity (FVC) (%) and modified Rodnan skin score (mRSS) values in 280 patients with systemic sclerosis (SSc) and diseases related to SSc. Numbers in bars, absolute counts. *P *values are results of the chi-square test. *For the chi-square test, because of the low case numbers in the last group, the last two mRSS groups were joined to hold the test assumptions (maximum 20% of cells have expected cell count < 5).

Among the 280 patients with SSc, anti-topo I-positive patients were characterized by fibrotic signs of SSc. As detected by LIA, anti-topo I-positive patients had a significantly higher prevalence of lung fibrosis (68.6%) when compared with the anti-topo I-negative group (23.3%). In addition, patients with anti-topo I antibodies had a lower predicted FVC when compared with the anti-topo I-negative SSc group (Tables 1 and 3). Only 15.6% of the patients with a predicted FVC > 85% had anti-topo I antibodies, but 55% of the patients with a FVC of 50% to 69% had anti-topo I antibodies (Figure 2). The association between the group of predictive FVC and the presence of anti-topo I antibodies was significant (*P *= 0.006).

Similar results were obtained for the predicted DLCO by a single breath. Patients with anti-topo I antibodies had a lower predicted DLCO when compared with antibody-negative patients (Table 3). Of the patients with a predicted DLCO > 85%, 15.5% were anti-topo I-positive; in contrast, the frequency of patients with anti-topo I antibodies increased to 43.8% in patients with DLCO < 35% (Figure 2). Again, the groups of DLCO levels were significantly related to the presence of anti-topo I antibodies (*P *< 0.001). Patients with anti-topo I antibodies also had a higher mRSS compared with patients without anti-topo I antibodies (Table 3). Only 2.2% of patients with a mRSS between 0 and 3 had anti-topo I antibodies. In contrast, 61.4% of the patients with a mRSS > 14 were anti-topo I antibody-positive (Figure 2). The higher the skin score, the higher the probability of being anti-topo I-positive (*P *< 0.001).

Patients with anti-topo I antibodies had a higher prevalence of tendon friction rubs that could be interpreted as a sign of active disease. Furthermore, anti-topo I antibodies were associated with contractures and electrocardiogram changes (Table 3).

### Anti-topoisomerase I-positive patients showed a higher disease activity and mortality

The SSc activity score was available for 266 out of 280 patients. Patients with anti-topo I antibodies as detected by LIA revealed a higher disease activity score when compared with antibody-negative SSc patients (Table 3). There was also a weak correlation between disease activity and signal strengths determined by LIA (*P *= 0.01, ρ = 0.234). The correlation was slightly better for patients with diffuse SSc. Here, the Spearman's rank correlation coefficient was 0.237 for the correlation between disease activity and LIA data (data not shown).

**Figure 3:**
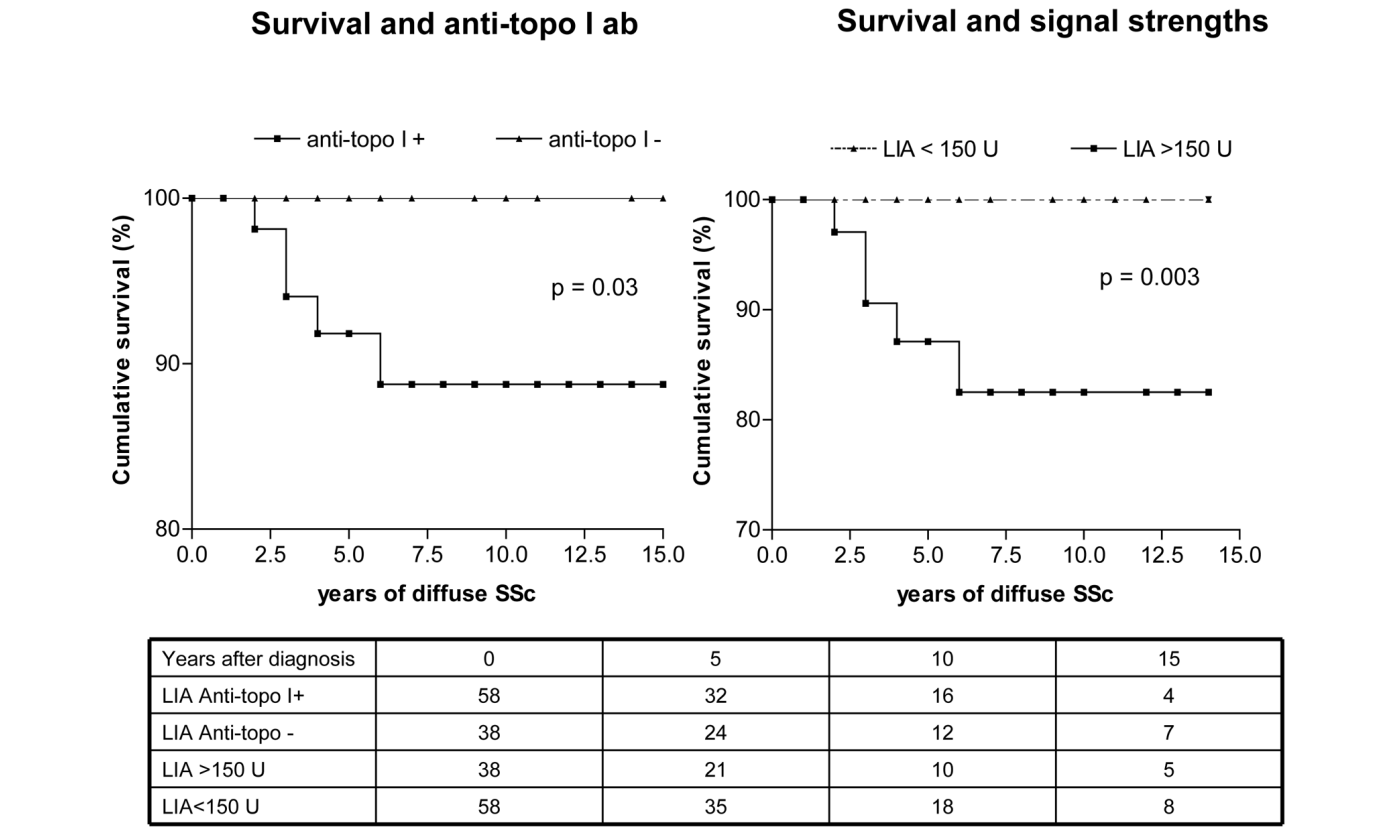
Cumulative survival from time of diagnosis related to the presence of anti-topoisomerase I antibodies. Cumulative survival rates from the time of diagnosis related to the presence of anti-topoisomerase I antibodies (anti-topo I ab) in our cohort of 96 patients with diffuse systemic sclerosis (SSc) as detected by line immunoblot assay (LIA). Signal strengths above 150 units were associated with increased mortality (*P *= 0.003).

After serum withdrawal for antibody detection, all patients were followed up for a mean period of 24.6 months. During this period, 16 SSc patients of our cohort died on average 7 years after diagnosis – 14 patients from SSc-associated complications, two patients from malignancies. In diffuse SSc patients, there was a low overall cumulative mortality. Eight patients of the diffuse SSc group died, and five of these patients were anti-topo I antibody-positive, revealing no significant differences between the anti-topo I-positive and anti-topo I-negative patients. The presence of anti-topo I antibodies was important for early mortality (*P *= 0.03; Figure 3a), however, especially when high signal strengths above 150 units were studied in the diffuse subset of SSc patients (*P *= 0.003; Figure 3b).

### Signal strengths in line immunoblot assay correlated with the extent of skin and lung fibrosis

LIA signals correlated to the extent of skin and lung fibrosis. There was a weak but significant correlation between the LIA signal strengths and mRSS values (*P *= 0.01, ρ = 0.413). Furthermore, the signal strengths correlated negatively with the predicted DLCO values by a single breath (*P *= 0.01, ρ = -0.219) and with the predicted FVC values (*P *= 0.01, ρ = -0.215).

## Discussion

The present study demonstrates the diagnostic significance of anti-topo I autoantibodies for clinical assessment of SSc patients in a large monocentric cohort. Antibody detection was performed by means of two commercial test systems, LIA and ELISA, both of which equally identified patients with active diffuse SSc with a higher burden of skin and lung fibrosis, electrocardiogram changes, digital ulcers, and contractures. Importantly, detected signal strengths correlated with skin score values, lung function parameters, and disease activity. We also showed a negative correlation between the predicted DLCO levels and the levels of anti-topo I antibodies, to our knowledge not identified by other studies, suggesting that the decline of DLCO in anti-topo I-positive patients is associated with lung fibrosis. Additionally, high signal strengths were associated with increased mortality in the diffuse SSc patients, indicating the potential of the applied test systems to identify patients with a more severe form of diffuse SSc.

Our cohort is part of the EUSTAR network and, compared with the EUSTAR patients, our anti-topo I-positive SSc patients showed similar clinical data, such as mean mRSS, age, disease duration, frequency of lung fibrosis, pulmonary arterial hypertension, and percentages of predicted DLCO [16]. In line with this correlation, the frequency of anti-topo I-positive patients was nearly identical – with 60.4% in our cohort compared with 60.8% in the EUSTAR cohort. For the EUSTAR database, the test used to identify anti-topo I antibodies is not defined and each centre is free to use the method most available. Our results support the conclusion that LIA or ELISA can be used similarly to other assays without loss of information. Furthermore, the similarities of our cohort with the European databank suggest that our cohort of SSc patients is representative for European SSc patients. Nevertheless, the frequency of anti-topo I antibodies is much higher than found in other cohorts, such as the Pittsburgh cohort with a frequency of 26% among diffuse SSc patients [6]. In their cohort, anti-topo I antibodies were detectable in 25% of the patients with limited SSc [1], representing a contrast to the low frequency of 6.2% in our 113 limited SSc patients.

In our monocentric cohort, most patients have suffered from SSc for longer than 6 years (Table 1). At the time of the present study, patients were already classified according to their maximal skin sclerosis. According to the DNSS and EUSTAR assessment strategy, the subclassification does not change – independent of the current distribution of skin sclerosis. Since LeRoy classification criteria are easily applicable to the extremes of limited SSc and diffuse SSc, there are patients between these typical SSc features. In those patients between these features, access to antibody levels, disease course, and organ manifestations could be helpful to classify the disease. We assume that the number of patients with current limited SSc is higher even in the anti-topo I antibody-positive patients. This could explain differences in the prevalence of anti-topo I antibodies among limited SSc patients. Furthermore, differences in the subclassification, antibody status, and clinical associations could be confounded by different ethnic backgrounds [11,29]. In our study, the population of anti-topo I-positive patients was almost homogeneously Caucasian. Only six patients in the anti-topo I-negative cohort had a different genetic background. By deleting these genetically different patients, the results of statistical analyses did not become different. Taking these factors together, in the present study the classification of the SSc patients into diffuse SSc and limited SSc was not done exclusively by measuring the current distribution of skin sclerosis. We also were aware of the autoantibody findings and the previous course of disease that can be critically discussed. A limitation of the present study is therefore a possible overestimation of diffuse SSc patients in our cohort. As recently published by our group analysing a large cohort of more than 1,000 German SSc patients, however, skin sclerosis is only of limited value to identify SSc patients with severe organ manifestations [30].

As shown by other studies using much larger cohorts, anti-topo I antibodies characterize patients with a higher extension of skin and lung fibrosis [1,6,13,16]. Other studies have also identified associations between the presence of anti-topo I antibodies with digital ulcers and with arthritis not confirmed by other works [6,30,31]. Furthermore, the assessment of the organ involvement seems to be crucial and often varies in different studies. When cardiac involvement is studied, different definitions are used. One group defined cardiac involvement by the presence of pericarditis or conduction disturbances of nonischaemic origin, and identified an association between cardiac involvement and the presence of anti-topo I antibodies – supporting the results from our study [5]. Another group defined heart involvement by symptomatic pericarditis, congestive heart failure, or arrhythmias requiring treatment and did not find any association with the serological status [6]. Since the assessment of subjective symptoms can be influenced by different reasons, and – as shown especially for the therapy of arrhythmias – guidelines and treatment modalities may vary, there is a need to use objective parameters to identify involvement of specific organs. International consensus criteria for assessment would be important for the evaluation of a potential biomarker and also to identify differences in the prevalence of organ involvement, but have so far not been established. In this setting, large monocentric cohorts with a homogeneous assessment could provide the most valuable data.

Comparing the cumulative survival in different studies, there was a high cumulative survival among our diffuse SSc patients and also among the anti-topo I-positive diffuse SSc patients in our cohort (about 92% in 5 years; Figure 3).

In an Italian cohort, cumulative 5-year survival from the first diagnosis was a little bit lower, with about 86% in anti-topo I-positive patients. The prevalence of organ involvement was similar to our group; however, by providing lung function or mRSS data, the extent of skin and lung fibrosis was not specified, making a comparison between the groups difficult [14]. In the Pittsburgh group, the cumulative 5-year survival from the initial visit of 102 anti-topo I-positive patients was about 70%. When the 68 diffuse anti-topo I-positive patients were studied, cumulative survival from the first symptom based on disease classification and autoantibody status was about 82% [6]. The high mean mRSS of 32 indicates an SSc cohort with severe SSc that seems to be not representative for cohorts found in Europe among the anti-topo I-positive patients, which was also suggested by other workers [32]. The EUSTAR database will provide the mortality found in SSc patients in Europe, providing a valuable tool to identify differences among the European countries and different ethnic groups.

For the evaluation of the diagnostic impact of a biomarker such as anti-topo I antibodies and for the comparison with other studies, the interval between antibody detection and clinical assessment may be important. Sato and colleagues also described a correlation between the levels of anti-topo I antibodies and mRSS values and the predicted vital capacities in a small number of anti-topo I-positive patients (n = 30) [30]. The correlations were much better than in our cohort. In contrast to our study, the authors did not provide information about the time point for the assessment of clinical data. For a progressive disease such as SSc, the degree of correlation between anti-topo I antibodies and the cumulative mRSS and FVC may be better if patients with more advanced disease were assessed. On the other hand, as shown by others, loss of anti-topo I reactivity during the course of the disease may occur in up to 20% of the patients [12], suggesting that a simultaneous assessment could be important.

Using the mRSS as a surrogate parameter to measure disease activity, other studies could also show an increased disease activity in anti-topo I-positive patients [3]. The mRSS, however, is only one parameter in the different disease activity scores used [17,18]. In the present study we have shown for the first time a weak but significant correlation between disease activity scores and the levels of anti-topo I antibodies or the signal strengths obtained by LIA or ELISA. For the assessment of the disease activity, we have used the EUSTAR activity score – suggesting that this method is a valuable test for the assessment of disease activity, as supported by Valentini and colleagues [33].

In conclusion, anti-topo I antibodies represent an appropriate and important parameter for the diagnosis and risk assessment of SSc patients. As exemplified here for the evaluation of anti-topo I antibodies, a precise characterization of patients and international consensus criteria for the assessment of patients are crucial and may influence the results. Where these tools are not available, large monocentric cohorts may provide a valuable tool for the evaluation of biomarkers.

## Conclusion

Anti-topo I autoantibodies can provide important information for the diagnosis of SSc patients. For the detection of these antibodies, carefully validated monospecific test methods, such as the LIA and ELISA applied in the present study, are equally competent and allow risk assessment due to the correlation between signal strength and severity of fibrotic manifestations, disease activity and prognosis.

The evaluation of potential biomarkers would profoundly benefit from the development and implementation of international consensus criteria for the classification of SSc patients. In this respect, large monocentric cohorts with a standardized assessment may provide the most valuable approach at present.

## Abbreviations

anti-topo I: anti-topoisomerase I; DLCO: predicted diffusion capacity; DNSS: Deutsches Netzwerk (German Network) of Systemic Scleroderma; ELISA: enzyme-linked immunosorbent assay; EUSTAR: European Scleroderma Trial and Research; FVC: predicted forced vital capacity; LIA: line immunoblot assay; mRSS: modified Rodnan skin score; SSc: systemic sclerosis.

## Competing interests

GR received lecturer's fees from EUROIMMUN AG to show the data at the 'Eurodoctor' meeting in Brussels. KH was invited by EUROIMMUN AG to participate in a national meeting to show the results of the study. After finishing the study, KH received a grant from EUROIMMUN AG for another scientific work. All the other authors declare that they have no competing interests.

## Authors' contributions

KH performed preclinical analyses, statistics, graphics and has partially written the manuscript. CD, AJ and WM developed the LIA and ELISA and performed the tests. CSB and MB provided the clinical data together with GR. MB corrected and helped to write the manuscript. DH helped with the statistics and guided KH to perform the right tests. KE and FH as well as GRB discussed the data with the last author and gave intellectual contributions. KE provided sera for the analysis. WS organized all cooperation with EUROIMMUN AG and provided intellectual contributions. GR, as the last and responsible author, initiated this study and controlled the work. GR wrote and reviewed the manuscript.
